# Supplementary material for: Resolving the full spectrum of human genome variation using Linked-Reads
Source: Genome Res. 2019 Apr;29(4):635–45. doi: 10.1101/gr.234443.118 (PMC6442396; doi:10.1101/gr.234443.118)
Supplement: Supplemental Material [file supp_gr.234443.118_Supplemental_longranger-2.2.zip › longranger-2.2.tar.bz2!/lr2.2-public/lib/stan/cmdstan/stan/doc/api/html/structstan_1_1prob_1_1include__summand.html]

Stan: stan::prob::include\_summand< propto, T1, T2, T3, T4, T5, T6, T7, T8, T9, T10 > Struct Template Reference


|  |  |
| --- | --- |
|  | Stan  2.5.0  probability, sampling & optimization |


- Main Page
- Related Pages
- Namespaces
- Classes
- Files

- Class List
- Class Index
- Class Hierarchy
- Class Members

All Classes Namespaces Files Functions Variables Typedefs Enumerations Enumerator Friends Macros Pages

- stan
- prob
- include\_summand

Public Types |
List of all members

stan::prob::include\_summand< propto, T1, T2, T3, T4, T5, T6, T7, T8, T9, T10 > Struct Template Reference

Template metaprogram to calculate whether a summand needs to be included in a proportional (log) probability calculation.
More...

`#include <traits.hpp>`

|  |  |
| --- | --- |
| Public Types | |
| enum | { value } |
|  | `true` if a term with the specified propto value and subterm types should be included in a proportionality calculation. More... |
|  | |

## Detailed Description

### template<bool propto, typename T1 = double, typename T2 = double, typename T3 = double, typename T4 = double, typename T5 = double, typename T6 = double, typename T7 = double, typename T8 = double, typename T9 = double, typename T10 = double> struct stan::prob::include\_summand< propto, T1, T2, T3, T4, T5, T6, T7, T8, T9, T10 >

Template metaprogram to calculate whether a summand needs to be included in a proportional (log) probability calculation.

For usage, the first boolean parameter should be set to `true` if calculating a term up to proportionality. Other type parameters should be included for all of the types of variables in a term.

The `value` enum will be `true` if the `propto` parameter is `false` or if any of the other template arguments are not constants as defined by `stan::is_constant<T>`.

Template Parameters
:   |  |  |
    | --- | --- |
    | propto | `true` if calculating up to a proportionality constant. |
    | T1 | First |

Definition at line 35 of file traits.hpp.

## Member Enumeration Documentation

template<bool propto, typename T1 = double, typename T2 = double, typename T3 = double, typename T4 = double, typename T5 = double, typename T6 = double, typename T7 = double, typename T8 = double, typename T9 = double, typename T10 = double>

anonymous enum

`true` if a term with the specified propto value and subterm types should be included in a proportionality calculation.

| Enumerator | |
| --- | --- |
| *value* |  |

Definition at line 42 of file traits.hpp.

---

The documentation for this struct was generated from the following file:

- src/stan/prob/traits.hpp


---

[ Stan Home Page ]

*© 2011–2014,
Stan Development Team.*
